# International Capacity‐Building Initiatives for National Bioethics Committees

**DOI:** 10.1002/hast.711

**Published:** 2017-05-22

**Authors:** Eugenijus Gefenas, Vilma Lukaseviciene

## Abstract

*During the last two decades, national bioethics committees have been established in many countries all over the world. They vary with respect to their structure, composition, and working methods, but the main functions are similar. They are supposed to facilitate public debate on controversial bioethical issues and produce opinions and recommendations that can help inform the public and policy‐makers. The dialogue among national bioethics committees is also increasingly important in the globalized world, where biomedical technologies raise ethical dilemmas that traverse national borders. It is not surprising, therefore, that the committees are established and active in the technologically advanced countries. There have also been a few international capacity‐building initiatives in bioethics that have had a dual task: networking among existing national bioethics committees and helping establish such committees in those countries that still lack them. The problem is that, due to a lack of information, it is not clear what problems and challenges committees face in the transitioning societies often characterized as low‐ and middle‐income countries*.

## Comparative Analyses

During the last two decades, national bioethics committees have been established in many countries all over the world. They vary with respect to their structure, composition, and working methods, but the main functions are similar. They are supposed to facilitate public debate on controversial bioethical issues and produce opinions and recommendations that can help inform the public and policy‐makers. The dialogue among national bioethics committees is also increasingly important in the globalized world, where biomedical technologies raise ethical dilemmas that traverse national borders. It is not surprising, therefore, that the committees are established and active in the technologically advanced countries.

There have also been a few international capacity‐building initiatives in bioethics that have had a dual task: networking among existing national bioethics committees and helping establish such committees in those countries that still lack them. The problem is that, due to a lack of information, it is not clear what problems and challenges committees face in the transitioning societies often characterized as low‐ and middle‐income countries.

## Capacity‐Building Initiatives

In Europe, the initiative to facilitate establishment and networking of national bioethics committees was started in 1992 by the European Conference of National Ethics Committees, known as COMETH, which was sponsored by the Council of Europe. COMETH was explicit about the goal of promoting cooperation among national bioethics committees. COMETH's activities included encouraging exchanges of information and the sharing of experience, developing a European database, carrying out studies on questions of common interest, organizing meetings at the European or regional level, helping countries that wanted a national ethics committee to set up and run one, and promoting public debate on ethical issues raised by progress in biology, medicine, and public health. After the last COMETH conference, which took place in 2007 in Berlin,^1^ the organization's activities were taken over by the National Ethics Committees Forum. This forum is sponsored by the European Commission and holds meetings of the European Union national bioethics bodies on a yearly basis. However, whereas COMETH provided opportunity for more than forty member states of the Council of Europe to bring together representatives of the national bioethics bodies, the European Union at present comprises only twenty‐eight member states. This means that representatives of so‐called transition countries (those in the process of transitioning to democratic governance and a market economy) of the Council of Europe (such as Albania, Armenia, Azerbaijan, Bosnia and Herzegovina, Georgia, Montenegro, the Republic of Moldova, the Russian Federation, Serbia, the former Yugoslav Republic of Macedonia, and Ukraine) that would also be categorized as low‐ or middle‐income countries by the World Bank do not have the opportunity to meet on a regular basis at the European forums.

This is why global initiatives to support capacity building and networking among national bioethics committees have become increasingly important. One of the most notable capacity‐building initiatives of this type has been the Global Summit of National Bioethics Advisory Bodies. The first such summit was held in 1996 in San Francisco as a joint initiative of the U.S. National Bioethics Advisory Commission, which had recently been appointed, and the French National Consultative Committee on Ethics. Since then, the summit has been supported by the secretariat at the World Health Organization and held biannually. The need to “increase participation of low and middle income countries in future Global Summit meetings” and the establishment of additional national ethics and bioethics committees, particularly in Africa and Asia, was identified as a priority by the participants of the 8th Global Summit, in 2010.^2^ The most recent summit, in July 2016 in Berlin, offered many networking opportunities, as it attracted an impressive number of participating countries—one hundred as compared to the eighteen at the 1996 summit.^3^

Another important capacity‐building initiative has been the Assisting Bioethics Committees Project of the United Nations Educational, Scientific, and Cultural Organization. This initiative follows directly from the 2005 UNESCO Universal Declaration on Bioethics and Human Rights—the only global document that discusses the establishment of national bioethics bodies.^4^ Article 19 of this document defines the main features and functions of “independent, multidisciplinary and pluralist ethics committees” to be established, promoted, and supported at the appropriate level in order to “assess scientific and technological developments, formulate recommendations and contribute to the preparation of guidelines on issues within the scope of this Declaration; foster debate, education and public awareness of, and engagement in, bioethics” [*sic*.].

The Assisting Bioethics Committees project seems to be a needed extension of the UNESCO activity in bioethics, as the majority of UNESCO member states do not have national bioethics committees. Nation states that have established committees as a result of the project are Côte d'Ivoire (as of 2002), Guinea (2007), Madagascar (2007), Togo (2007), Gabon (2008), Kenya (2008), Colombia (2009), El Salvador (2009), Ghana (2009), Jamaica (2009), Mali (2009), Oman (2009),^5^ Malaysia (2010), Chad (2011), Malawi (2011), the Dominican Republic (2014), Ecuador (2014), and Comoros (2015). The establishment of new national bioethics committees and facilitation of their networking are commendable initiatives. The discussions at the 2016 Global Summit clearly showed that these global forums need to address problems and developments that extend across national borders, such as outbreaks of disease and the emergence of biotechnologies, as well as the effective use of public outreach and communications mechanisms by national bioethics committees—for example, how to raise social awareness of bioethical issues through education and media. However, some questions about the very creation of the committees must also need discussion. What are the real incentives to establish national bioethics committees in low‐ and middle‐income countries? It is generally unclear if and how bioethical concerns are discussed at the national level in the developing world, so how will we know if newly established committees of this type are fulfilling the tasks envisaged in article 19 of the Universal Declaration on Bioethics and Human Rights? It is important to ensure that the new institutions are not established merely to comply formally with the recommendation to create a national committee but, rather, that they satisfy genuine needs to deal with urgent and country‐specific bioethical concerns.

A study of national ethics committees conducted as a part of the project Stakeholders Acting Together on the Ethical Impact Assessment of Research and Innovation (SATORI project), which received funding from the European Commission's Seventh Framework Programme, can serve as a useful starting point to identify problems faced by national bioethics committees. The study was based on interviews with representatives from nongovernmental bioethics bodies, such as the United Kingdom's Nuffield Council on Bioethics, as well as governmental entities, such as the United States’ Presidential Commission for the Study of Bioethical Issues and several European bodies (including those from Austria, Denmark, Finland, France, Germany, Slovenia, Spain, and Serbia).^6^ One of the main challenges national bioethics committees face is developing better ways of reaching out to the public. The SATORI report also noted that the committees have given little attention to new technologies and the effects of their emergence. Finally, the study showed that the committees’ activities sometimes raise societal expectations that this kind of institution cannot fulfill. For example, the committees’ reports are sometimes criticized for not having a measurable impact, not being directly implementable, or not offering definitive solutions to problems—impossible standards, given that the committees are not juridical bodies and the problems they are addressing often have no clear‐cut solutions.

## Challenges in Low‐ and Middle‐Income Countries

Although the difficulties described above are probably shared by national bioethics committees around the world, some issues are particularly acute in low‐ and middle‐income countries. First, unlike with the committees described in the SATORI study, information about committees in low‐ and middle‐income countries is often missing. Even basic up‐to‐date information about the structure (such as affiliation, criteria for membership and composition, functions and mandate, and availability of the secretariat) and output (opinions and recommendations, annual reports, and so on) is very often not available on the web pages of the committees referred to in the UNESCO Global Ethics Observatory, a system of databases,^7^ or there are no active links to the websites of national bioethics committees in this system. This asymmetry between committees in developing and developed countries is probably at least partly a consequence of the limited resources available for the committees in less wealthy countries.

There could also be some more structural challenges. The lack of information and absence of web pages could reflect obstacles to developing relevant activities, such as engagement in a pluralistic discourse on bioethical issues, which has been one of the most important features of national bioethics committees. This is a rather likely scenario in authoritarian societies as well as in countries that are still in transition to democratic governance or are dominated by a single religious perspective. To give an extreme example, research that would reveal information about significant public health threats can be heavily suppressed—and the researchers imprisoned; this recently happened in Belarus, with research on the Chernobyl disaster coming under attack.^8^

Another structural difference between national bioethics committees in affluent countries and those in low‐ and middle‐income countries has to do with different conceptualizations of what problems are to be regarded as “ethical issues” and how issues should be prioritized. In some low‐ and middle‐income countries, for example, questions about technological developments are overshadowed by problems of corruption and scarcity of resources. It seems, therefore, that the socioeconomic and cultural context should shape the content and priorities of the committees’ activities. Capacity‐building initiatives should take this into account. National bioethics committees’ activities cannot be easily modeled on examples from wealthy countries because both the organizational features of these bodies and the issues to be included into their agendas are shaped by socioeconomic realities. This could be one reason that global approaches to bioethics based on a universal set of principles appear to be of a limited practical value.^9^

## The Diversity of European Committees

With significant variance in size, social conditions, and income levels among the nations of Europe, the national bioethics committees in the region must function in many different socioeconomic contexts. An attempt to collect information on committees within the European Union reveals an asymmetry between long‐time member states and the thirteen countries that joined the union after 2004. Our efforts to collect data on committees from both these long‐time and new member states included examining information in the UNESCO Global Ethics Observatory and searching the Internet for information about the institutions listed as National Ethics Councils Forum members in the Register of European Commission expert groups.^10^ We found that information about national bioethics committees in the newer member states is limited, as it is for such committees in many low‐ and middle‐income countries around the world. While all the committees in the long‐time E.U. member states provide comprehensive information on the Internet about their structure and activities—in most cases, also offering information in English—information about the activities of the new member states’ committees is comparatively limited: not all of them have web pages, and only a few of those who have websites present information in English.

European committees also differ with regard to structure and output. The committees in the old E.U. countries usually act only as advisory institutions, issuing opinions or recommendations on emerging bioethical controversies, while committees in the newer member states are often also involved in the review of research on human subjects. For example, Slovenia's National Medical Ethics Committee reviews most of the country's biomedical research, the Lithuanian Bioethics Committee reviews multicenter studies, and some projects related to the human genome are examined by the Latvian Central Medical Ethics Committee. Arguably, committees that incorporate the functions of research ethics committees do not comply with the goals of national ethics advisory bodies emphasized in the documents used by different capacity‐building initiatives. However, it could also be argued that broadening the scope of the committees’ functions, particularly in small countries with rather limited means allocated to bioethics, could be a way to institutionalize the committees and strengthen their status on the national level.

The complexity of issues related to the establishment and functioning of national bioethics committees in low‐ and middle‐income countries requires a more comprehensive analysis, but some preliminary suggestions can still be made. One of the priorities of international capacity‐building initiatives could be to close the informational gap between affluent countries and low‐ and middle‐income countries. National bioethics committees, particularly those established as a result of international initiatives, should be encouraged to make information about their structure and functions more easily available. This would allow for a better understanding of the challenges these committees face, and it might help the committees to deal with the obstacles and facilitate the development of bioethics in these countries. Global capacity‐building initiatives should also be more explicit about the different challenges faced by the committees operating in different sociocultural contexts. Perhaps committees in different contexts should be able to adopt different modalities and pursue different strategies to develop bioethical discourse.
